# Association of P2Y_2_ receptor SNPs with bone mineral density and osteoporosis risk in a cohort of Dutch fracture patients

**DOI:** 10.1007/s11302-012-9326-3

**Published:** 2012-07-08

**Authors:** Anke Wesselius, Martijn J. L. Bours, Zanne Henriksen, Susanne Syberg, Solveig Petersen, Peter Schwarz, Niklas R. Jørgensen, Svenhjalmar van Helden, Pieter C. Dagnelie

**Affiliations:** 1 Department of Epidemiology, School for Public Health and Primary Care (CAPHRI), Maastricht University, Peter Debyeplein 1, P.O. Box 616, 6200 MD Maastricht, The Netherlands; 2 Research Center for Ageing and Osteoporosis, Departments of Clinical Biochemistry and Medicine, Copenhagen University Hospital Glostrup, Glostrup, Ndr Ringvej 57-59, 2600 Glostrup, Denmark; 3 Department of Trauma Surgery Isala Clinics, Zwolle; formerly Department of Trauma Surgery, Maastricht University Medical Centre, P.O. Box 5800, 6202 AZ Maastricht, The Netherlands

**Keywords:** P2Y_2_ receptor, Osteoporosis, Bone mineral density, Polymorphisms

## Abstract

The P2Y_2_ receptor is a G-protein-coupled receptor with adenosine 5′-triphosphate (and UTP) as natural ligands. It is thought to be involved in bone physiology in an anti-osteogenic manner. As several non-synonymous single nucleotide polymorphisms (SNPs) have been identified within the P2Y_2_ receptor gene in humans, we examined associations between genetic variations in the P2Y_2_ receptor gene and bone mineral density (BMD) (i.e., osteoporosis risk), in a cohort of fracture patients. Six hundred and ninety women and 231 men aged ≥50 years, visiting an osteoporosis outpatient clinic at Maastricht University Medical Centre for standard medical follow-up after a recent fracture, were genotyped for three non-synonymous P2Y_2_ receptor gene SNPs. BMD was measured at three locations (total hip, lumbar spine, and femoral neck) using dual-energy X-ray absorptiometry. Differences in BMD between different genotypes were tested using analysis of covariance. In women, BMD values at all sites were significantly different between the genotypes for the Leu46Pro polymorphism, with women homozygous for the variant allele showing the highest BMD values (0.05 > *p* > 0.01). The Arg312Ser and Arg334Cys polymorphisms showed no differences in BMD values between the different genotypes. This is the first report that describes the association between the Leu46Pro polymorphism of the human P2Y_2_ receptor and the risk of osteoporosis.

## Introduction

Osteoporosis is a condition of the skeleton characterized by decreased bone density and bone structure changes, reducing its strength and resulting in increased risk of fragility fractures. This bone disease is one of the most common health problems among elderly in the Western society. In Europe, approximately 30 % of women and 12 % of men aged over 50 suffer from osteoporosis [1].

Due to the growing elderly population as well as the sedentary lifestyle and changes in nutritional habits, the incidence of osteoporosis and related fractures is expected to increase exponentially worldwide over the next decades. In terms of human capital losses as well as use of health care resources, this will lead to an enormous social economical burden. The annual cost of osteoporotic fractures nowadays is suggested to be approximately 36 billion euros [2] and is anticipated to increase to 106 billion euros in 2050 (www.iofbonehealth.org).

Bone remodeling is the process responsible for maintaining bone quality during life. In this process, bone tissue is continuously renewed by tight regulation of the balance between bone resorption by osteoclasts and bone formation by osteoblasts [3]. The underlying mechanisms responsible for regulating the balance between bone resorption and formation are currently not fully understood. However, it has become more and more clear that purinergic signaling plays an essential role in this process [4]. Purinergic signaling is dependent on binding of nucleotides to specific so-called purinergic receptors. These purinergic receptors can be divided in two major receptor subclasses, namely, P1 and P2 receptors, according to their distinct affinities for adenosine and ATP/ADP, respectively [5]. The P2 receptor subclass can be further subdivided in P2X receptors, of which seven subtypes are known (P2X_1, 2, 3, 4, 5, 6, 7_), and P2Y receptors, of which eight subtypes are known (P2Y_1, 2, 4, 6, 11, 12, 13, 14_) [6]. Expression of several of the P2X and P2Y receptor subtypes by different bone cell types has been demonstrated (reviewed in [7]).

The P2Y_2_ receptor is fully activated by ATP and UTP [8] and is expressed in a variety of tissues in the human body where it plays important roles in various processes by activation of phospholipase C β and subsequent generation of inositol (1, 4, 5)-trisphosphate, which leads to a rise in calcium and activation of protein kinase C [9]. The expression of the P2Y_2_ receptor on human bone cells was first reported by Bowler et al. [10], who found that P2Y_2_ receptor DNA was expressed by human osteoblasts derived from bone explants. This finding was later confirmed by another group [11]. Osteoblast-like cells from the human osteosarcoma cell lines MG-63 and OHS-4 have also been shown to express the P2Y_2_ receptor [12]. In addition to expression by osteoblasts, expression of the P2Y_2_ receptor on osteoclasts has been observed in osteoclasts derived from a human giant cell tumor of a bone [10].

Different authors have shown that the P2Y_2_ receptor is involved in the bone biology [13–15]. In vitro experiments showed that ATP and UTP at concentrations of 1–10 μM, but not adenosine and ADP, strongly inhibited mineralized bone nodule formation by cultured rat osteoblasts [13], which was suggested to involve either P2Y_2_ or P2Y_4_ receptors. Later studies demonstrated that a P2Y_4_ antagonist failed to prevent the above nucleotide-induced inhibition of mineralization, suggesting that activation of the P2Y_2_ receptor was responsible for the observed functional effects of ATP and UTP on osteoblast-mediated bone formation [16]. In agreement with these findings, Orriss and colleagues [15] showed that ATP and UTP (but not ADP and UDP) inhibited the expression and activation of alkaline phosphatase. Animal studies using a P2Y_2_ knock-out model confirmed the putative anti-osteogenic effect of the P2Y_2_ receptor. Mice lacking the P2Y_2_ receptor showed a 9 % increase in bone mineral content of the femora and a 17 % increase in BMC of the tibiae compared to wild-type mice [15]. However, in contrast to these anti-osteogenic findings, Katz and colleagues [17] showed in osteoblastic cells up-regulation of the P13/Akt signal transduction pathway by extracellular ATP, which is important in growth and survival of osteoblasts [18]. Since both the P2Y_2_ agonists ATPγS and UTP showed increased Akt phosphorylation, the authors suggested that the P2Y_2_ receptor is responsible for this P13/Akt up-regulation, leading to osteoblastic proliferation.

The above data make the P2Y_2_ receptor gene a possible candidate gene in the causation of osteoporosis. Based on this, we hypothesized that single nucleotide polymorphisms (SNPs) in the P2Y_2_ receptor resulting in aberration of receptor function would affect bone mineral density (BMD) in humans.

**Fig. 1:**
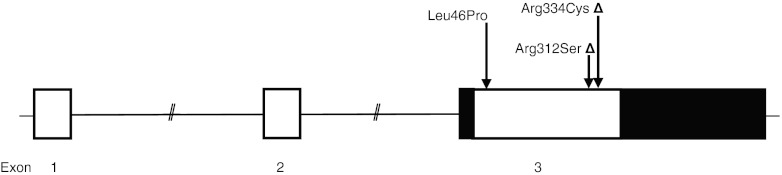
Investigated P2Y_2_ receptor polymorphisms. *Triangle* polymorphisms with increased receptor function

Dasari et al. (1996) first mapped the P2Y_2_ receptor gene to the human chromosome 11q13.5-14.1, spanning over 18 kb coding for 377 amino acids and consisting of three exons separated by two introns (Fig. 1). This receptor is known to have three splice variants. To date, over 100 SNPs have been reported within the P2Y_2_ receptor gene according to the international Hapmap project, of which at least three non-synonymous SNPs lead to an amino acid change (Fig. 1). Two of these three non-synonymous SNPs have known effects on P2Y_2_ receptor function, leading to a gain-of-receptor function [19, 20]. For the Arg334Cys polymorphism, it was shown that carriers of the 334Cys mutation produced slower accumulation of intracellular inositol triphosphates and had a significantly increased transient Ca^2+^ influx compared to wild-type [19, 20]. Subjects homozygous for the Arg312Ser polymorphism showed an increased transient Ca^2+^ influx compared to wild-type cells [20]. The functional effect of the other non-synonymous SNP (Leu46Pro) has not yet been established.

In the present study, we investigated the presence and frequency of three non-synonymous P2Y_2_ gene polymorphisms in a Dutch cohort of fracture patients and analyzed whether genetic variation in this purinergic receptor was associated with altered BMD, i.e., osteoporosis risk. We chose a fracture cohort as this is characterized by the high prevalence of osteoporosis [21].

## Methods

### Study population and design

The study base for the present study consisted of men and women aged ≥50 years, who visited an osteoporosis outpatient clinic at the Maastricht University Medical Centre (MUMC^+^), the Netherlands, for standard medical care following a recent fracture. Fracture patients suffering from a disease of bone metabolism other than osteoporosis (e.g., Paget disease, bone tumors, and hyperparathyroidism) were excluded from participation.

The study was approved by the ethical committee of the University Hospital Maastricht and Maastricht University, and all participants signed written informed consent after having received proper information about the study before performing any of the study procedures. Participants for the present study were recruited at the osteoporosis outpatient clinic at MUMC^+^ among patients receiving regular medical follow-up for a recent fracture. The regular medical follow-up procedure for fracture patients was as follows [21]:

1. Patients, who presented with a clinical fracture at the emergency unit or were hospitalized because of a fracture, were invited to the fracture and osteoporosis outpatient clinic;
2. During a first consultation, usually 2–6 weeks following the fracture, besides receiving information about the outpatient clinic and possible treatment regimes, patients were asked to undergo a bone densitometry;
3. During a second consultation, usually 2–4 weeks later, BMD measurement was performed by dual X-ray absorptiometry (DXA) and, in addition, risk factors for falls and osteoporosis were assessed; if indicated, medicinal treatment for osteoporosis was started according to the Dutch osteoporosis guideline recommendation.

For the present study, we recruited subjects from the above-mentioned population of fracture patients by using two different procedures: first, between August 2008 and December 2009, blood was collected from patients who visited the osteoporosis outpatient clinic. All patients received extensive oral and written information about the study during their first consultation; then, during their second consultation, written informed consent was obtained, and blood samples were collected and stored at −80 °C for subsequent DNA extraction and genotyping.

Second, to increase statistical power, saliva was collected from fracture patients who had formerly visited the osteoporosis outpatient clinic before August 2008. Eligible patients for this recruitment procedure were identified using an existing patient database of the osteoporosis outpatient clinic at MUMC^+^, which had been initiated in September 2004. All eligible patients received an information package by mail, which included (1) a letter to inform patients about the present study, (2) a standard device to collect saliva together with instructions for its use, (3) an informed consent form, and (4) a return envelope with pre-printed address. Patients willing to participate were asked to sign the informed consent form, to donate a small amount of saliva, and to send both of these back to us in the return envelope. Patients, from whom no reaction was received within 2 weeks after the information package had been sent, were contacted once by telephone to increase the response rate.

### DNA extraction

#### Blood samples

DNA was extracted from blood in an automated procedure using Maxwell 16 DNA purification kits on the Maxwell 16 instrument (Promega, Madison, WI); 400 μl of blood collected in EDTA tubes were used and the isolation procedure was performed according to the manufacturer's instructions.

#### Saliva samples

For collection of a small amount of saliva for DNA extraction, we used a Salivette^TM^ (Sarstedt AG & Co. Numbrecht, Germany), a plastic vial containing a small cotton roll that needs to be chewed on for 45–60 s, yielding approximately 1.5 ml of saliva and is then placed back into the plastic vial. Patients were asked to use the Salivette at least 30 min after eating, drinking, or use of oral medication. Upon return, the Salivette^TM^ containing the saliva swab was stored in a refrigerator at 4 °C until DNA extraction. First, the swab kept in the collection tube was centrifuged at 4,000 rpm for 10 min, and the saliva was transferred to a 15-ml Nunc tube which was kept at 5 °C overnight. Using a pair of sterile tweezers, the swab was then transferred from the collection tube to a 50-ml Nunc tube; 4 ml sterile water was added and the tube was kept at room temperature overnight. The next day, the swab plus water was transferred back into the collection tube and again centrifuged at 4,000 rpm for 10 min; the saliva yield was again transferred to the 15-ml Nunc tube already containing the saliva yield from the day before. Next, cells were isolated from the saliva by centrifuging the saliva-containing 15-ml Nunc tube at 4,000 rpm for 10 min. Subsequently, the supernatant was carefully removed, leaving 600–800 μl over the pellet. DNA extraction was then carried out using Maxwell 16 DNA purification kits on the Maxwell 16 instrument (Promega, Madison, WI) according to the manufacturer's instructions.

### Genotyping

The study population was genotyped for three non-synonymous SNPs within the P2Y_2_ receptor gene that were selected based on their previously published functional effects on the P2Y_2_ receptor or were found in the dbSNP database for non-synonymous SNPs (Fig. 1). Genotyping was done by Sequenom (Sequenom, Hamburg, Germany) using the Sequenom MassARRAY ® iPLEX Gold assay, which uses PCR amplification followed by a single base pair primer extension reaction, resulting in an allele specific difference in mass between extension products. This mass difference allows the data analysis software to differentiate between SNP alleles using matrix-assisted laser desorption/ionization time-of-flight mass spectrometry.

### Internal validation study

To assess the accuracy of the genotyping assay, an internal validation study was performed in which a randomly selected number of samples (*N* = 45) were genotyped a second time, using restriction enzyme digestion of appropriate PCR products or Taqman assay. This was done according to a previously published protocol (Hansen et al. 2008). When the results were compared with the original genotyping, we observed a discrepancy between the two different genotyping methods of ~4.2 %. The discrepancy appeared to be smaller (~2.7 %) if the original genotyping with the Sequenom MassARRAY ® iPLEX Gold assay had failed for maximum of one SNP. Therefore, all subjects in whom the original genotyping had failed for at least two SNPs were excluded from statistical analysis.

### Bone density measurements

As part of the standard medical follow-up of fracture patients, BMD (in grams per cubic centimeter) of the lumbar spine (L2–L4), femoral neck, and total hip (trochanter and neck) was assessed by DXA, using the cross-calibrated Hologic QDR 4500 Elite densitometer (Waltham, Massachusetts, USA). This was usually done 4–10 weeks following the fracture, before starting medicinal treatment in case osteoporosis was diagnosed. BMD *T* score values were used to establish the presence or absence of osteoporosis (*T* ≤ −2.5) and osteopenia (−2.5 < *T* < −1).

### Statistical analysis

Distribution of genotype frequencies was tested for Hardy–Weinberg equilibrium (HWE) by chi-square test. Descriptive statistics were used to determine the prevalence of osteoporosis and osteopenia in the cohort of fracture patients, as well as to assess distributions of possible risk factors, including sex, age (in years), body mass index (BMI, in kilograms per square centimeter), previous fracture (yes/no), and family history of fractures (yes/no), which were all recorded during a patient's second consultation at the osteoporosis outpatient clinic. Furthermore, descriptive statistics were used to describe the occurrence of different fracture types.

Differences in BMD between the genotypes for each individual SNP were tested for significance by using the general linear model procedure for covariance analysis (ANCOVA) after testing for normal distribution of the data and uniformity of variances. Preliminary analyses showed that only sex, age, and BMI were associated with several SNPs. Therefore, all analyses were stratified by sex and adjusted for age and BMI.

The frequency of all possible haplotype combinations was investigated, and differences in BMD between the most frequent haplotype combinations were tested for significance using the general linear model procedure for ANCOVA. As a confirmatory approach, we used proportional odds logistic regression to estimate the influence of P2Y_2_ receptor genotypes on the odds of a low BMD *T* score value and thus on osteoporosis risk. For this approach, quintiles of the population were defined based on BMD *T* score values. The proportional odds assumption was tested using the chi-square score test.

For all analyses, *p* values < 0.05 were considered statistically significant. All analyses were performed using SAS, version 9.1.

## Results

### Study population

**Fig. 2:**
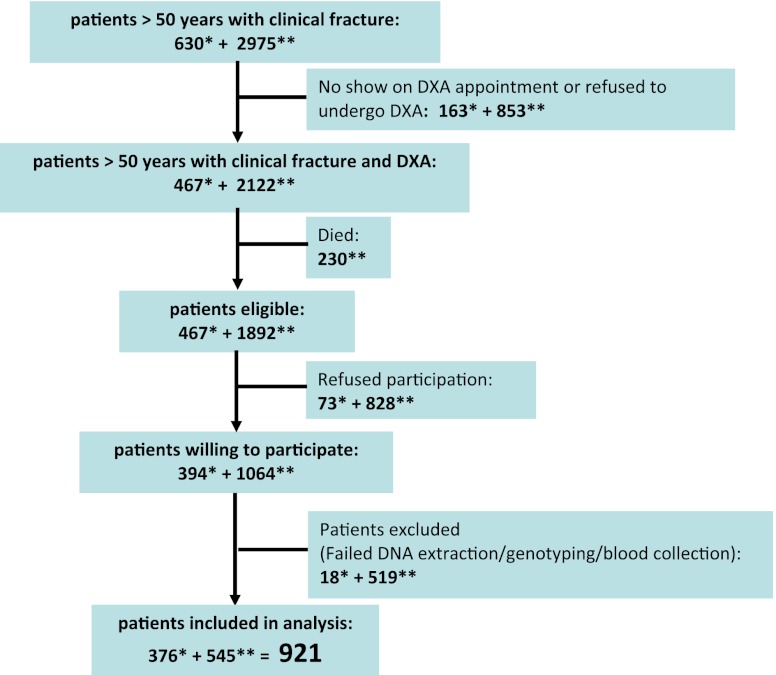
Flow chart of patient recruitment for the present study. *Single asterisk* number of patients recruited during phase 1 between August 2008 and December 2009. *Double asterisks* number of patients recruited during phase 2 between January and July 2010

Of the 630 patients with a recent fracture, who were invited to the osteoporosis outpatient clinic between August 2008 and December 2009, 467 (74.1 %) were willing to undergo a bone densitometry. Of these fracture patients, during their second consultation at the osteoporosis outpatient clinic, 394 (84.4 %) were willing to donate blood. The collection of blood failed for 13 (3.3 %) patients and genotyping for 5 (1.3 %) patients (Fig. 2), leaving 376 patients included for analysis.

Of the 2,975 fracture patients who had formerly visited the osteoporosis outpatient clinic between September 2004 and August 2008, 2,122 (71.3 %) had undergone a bone densitometry. Two hundred thirty of these patients had died in the meantime (10.8 %). Of the 1,892 former fracture patients who were invited by mail to participate in the present study, 1,064 (58.2 %) gave consent and returned saliva samples. DNA extraction failed for 27 (2.5 %) samples and genotyping for 492 (46.2 %) samples (based on the outcome of an internal validation study) (Fig. 2), leaving 545 patients included for analysis.

Characteristics of the 921 participants are listed in Tables 1 and 2. The final study population consisted of 690 women aged 65.5 ± 9.8 years (mean ± SD) and 231 men aged 63.5 ± 9.6 years. The prevalence of osteoporosis was 32.2 % among women and 26.4 % among men, and the prevalence of osteopenia was 48.0 % among women and 42.0 % among men. Hip fractures and fractures of the humerus were most common among subjects suffering from osteoporosis (11.8 and 15.7 %, respectively), whereas other common osteoporotic fractures, i.e., fractures of the lumbar spine and wrist, were more frequent in subjects suffering from osteopenia (4.8 and 29.7 %, respectively). No differences were observed between the data collected during the two recruitment procedures. Furthermore, no differences in baseline characteristics were observed between subjects included in the analyses and subjects excluded based on the internal validation study.

**Table 1:** Characteristics of the study population

	Total (*N* = 921) mean (SD)	Men (*N* = 231) mean (SD)	Women (*N* = 690) mean (SD)
Age (years)	65.0 (9.8)	63.5 (9.6)	65.5 (9.8)
Weight (kg)	72.5 (13.8)	82.29 (12.4)	69.2 (12.6)
Height (cm)	165.8 (9.1)	175.7 (7.3)	162.5 (6.9)
BMI (kg/m^2^)	26.3 (4.2)	26.6 (3.7)	26.2 (4.4)
Femoral neck BMD (g/cm^2^)	0.69 (0.13)	0.76 (0.13)	0.66 (0.12)
Total hip BMD (g/cm^2^)	0.84 (0.15)	0.95 (0.15)	0.80 (0.13)
Lumbar spine BMD (g/cm^2^)	0.93 (0.17)	0.98 (0.17)	0.91 (0.17)

*BMI* body mass index, *BMD* bone mineral density

**Table 2:** Prevalence of osteoporosis and osteopenia in the study population, and distribution of fractures

	Osteoporosis^a^	Osteopenia^b^	Normal BMD
	% (*N*)	% (*N*)	% (*N*)
Total study population (*N* = 921)	30.7 (283)	46.5 (428)	22.8 (210)
Men (*N* = 231)	26.4 (61)	42.0 (97)	31.6 (73)
Women (*N* = 690)	32.2 (222)	48.0 (331)	19.8 (137)
Type of fracture			
Humerus (*N* = 108)	15.7 (40)	11.6 (46)	11.2 (22)
Femur (*N* = 72)	11.8 (30)	8.3 (33)	4.6 (9)
Lumbar spine (*N* = 38)	4.3 (11)	4.8 (19)	4.1 (8)
Wrist (*N* = 206)	24.8 (63)	29.7 (118)	12.7 (25)
Other fracture (*N* = 424)	43.3 (110)	45.6 (181)	67.5 (133)

^a^Osteoporosis defined by BMD *T* score values, *T* ≤ −2,5

^b^Osteopenia defined by BMD *T* score values, −2.5 < *T* < −1

### P2RY_2_ genotypes

Minor allele frequency and information on Hardy–Weinberg equilibrium of the three genotyped non-synonymous SNPs within the P2Y_2_ receptor gene are shown in Table 3. The Arg312Ser was found to be in HWE, but both Leu46Pro and Arg334Cys showed significant deviation from HWE (*p* < 0.05).

**Table 3:** List of P2Y_2_ receptor SNPs for which the study population was genotyped

rs number	Base change	Polymorphism	MAF	HWE *p* value	Effect
rs2511241	137C > T	Leu46Pro	0.09	0.012	n.a..
rs3741156	936G > C	Arg312Ser	0.23	0.120	Gain-of-receptor function
rs1626154	1000C > T	Arg334Cys	0.16	0.031	Gain-of-receptor function

*MAF* minor allele frequency, *HWE* Hardy–Weinberg equilibrium, *n.a.* not available (no data published on functional effects of this polymorphism)

### Association of P2Y_2_ receptor genotypes with bone mineral density

**Fig. 3:**
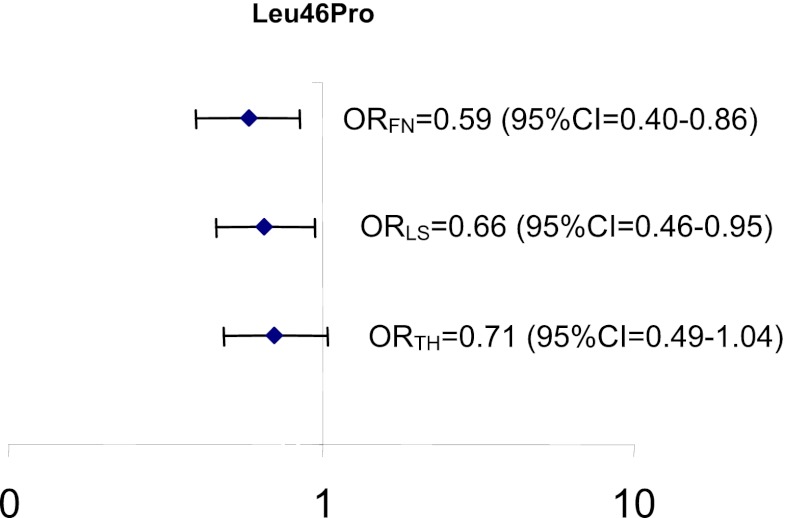
Graphical display of the risk on low BMD *T* score value of women carrying at least one wild-type allele of the Leu46Pro polymorphism (TT and TC genotypes) compared to women homozygous for the variant allele (CC genotype) at the total hip (*TH*), lumbar spine (*LS*), and femoral neck (*FN*)

In women, BMD values at the lumbar spine and femoral neck were significantly different between the genotypes for the Leu46Pro polymorphism, with women homozygous for the variant allele showing the highest BMD values (*p* = 0.03 and 0.01, respectively). BMD values at the total hip were borderline significantly higher in women homozygous for the variant allele (*p* = 0.05) (Table 4). The proportional odds logistic regression confirmed these results by showing that the odds of a lower *T* score (i.e., the risk of osteoporosis) was significantly decreased by approximately 30–40 % in women homozygous for the variant allele compared to the other two genotypes at the lumbar spine and femoral neck (lumbar spine OR = 0.66 [95 % CI, 0.46–0.95]; femoral neck OR = 0.59 [95 % CI, 0.40–0.86]) (Fig. 3). Although, both the Arg312Ser and Arg334Cys polymorphisms showed decreased BMD values in women homozygous for the variant allele, none of these differences were statistically significant. In men, no association between the different genotypes of each SNP and BMD values could be observed.

**Table 4:** BMD values for the individual genotypes for each single SNP

SNP	WT	HET	HOMO	*p* value^a^
Woman				
Leu46Pro	TT	TC	CC	
*N*	569	100	11	
BMD TH (g/cm^2^)	0.79 (0.13)	0.81 (0.14)	0.83 (0.13)	0.054
BMD LS (g/cm^2^)	0.90 (0.16)	0.93 (0.17)	0.99 (0.17)	0.026
BMD FN (g/cm^2^)	0.66 (0.12)	0.68 (0.12)	0.70 (0.14)	0.013
Arg312Ser	GG	GC	CC	
*N*	398	236	41	
BMD TH (g/cm^2^)	0.80 (0.13)	0.81 (0.14)	0.76 (0.13)	0.073
BMD LS (g/cm^2^)	0.91 (0.17)	0.91 (0.16)	0.90 (0.18)	0.926
BMD FN (g/cm^2^)	0.66 (0.11)	0.67 (0.12)	0.65 (0.11)	0.352
Arg334Cys	GG	GA	AA	
*N*	483	157	23	
BMD TH (g/cm^2^)	0.80 (0.13)	0.80 (0.14)	0.78 (0.13)	0.844
BMD LS (g/cm^2^)	0.91 (0.17)	0.91 (0.17)	0.90 (0.16)	0.947
BMD FN (g/cm^2^)	0.67 (0.11)	0.66 (0.12)	0.64 (0.12)	0.843
Men				
Leu46Pro	TT	TC	CC	
*N*	188	39	3	
BMD TH (g/cm^2^)	0.94 (0.15)	0.98 (0.15)	0.90 (0.12)	0.479
BMD LS (g/cm^2^)	0.98 (0.17)	1.00 (0.17)	0.88 (0.13)	0.429
BMD FN (g/cm^2^)	0.76 (0.13)	0.77 (0.13)	0.71 (0.10)	0.775
Arg312Ser	GG	GC	CC	
*N*	145	67	15	
BMD TH (g/cm^2^)	0.95 (0.16)	0.96 (0.14)	0.88 (0.16)	0.732
BMD LS (g/cm^2^)	0.97 (0.16)	1.00 (0.19)	0.97 (0.16)	0.462
BMD FN (g/cm^2^)	0.76 (0.13)	0.76 (0.13)	0.73 (0.14)	0.960
Arg334Cys	GG	GA	AA	
*N*	153	63	8	
BMD TH (g/cm^2^)	0.95 (0.14)	0.93 (0.17)	1.04 (0.16)	0.158
BMD LS (g/cm^2^)	0.99 (0.17)	0.95 (0.16)	1.00 (0.17)	0.272
BMD FN (g/cm^2^)	0.76 (0.13)	0.75 (0.14)	0.79 (0.16)	0.863

*p* values are shown for statistical analysis of covariance (ANCOVA) for the bone mineral density (BMD) parameters adjusted for age and BMI. Numbers are means (SD)

*LS* lumbar spine, *FN* femoral neck, *TH* total hip

^a^Adjusted for age and BMI

### Association of P2Y_2_ receptor haplotypes with bone mineral density

The seven most common haplotype combinations were tested for association with bone mineral density, that is TGG/TGG (H1), TGG/TCG (H2), TGG/TGA (H3), TGG/CGG (H4), TGG/TCA (H5), TGG/CCG (H6), and TCG/TCG (H7). This analysis included 591 women and 200 men and covered over 93 % of the total population. As shown in Table 5, in both women and men, the wild-type combination of the three SNPs (i.e., wild-type for each of the three polymorphisms, TGG/TGG) was the most frequent haplotype (31.2 and 34.0 %, respectively).

**Table 5:** BMD values for the different haplotype combinations

Genotype^a^	TGG/TGG (H1)	TGG/TCG (H2)	TGG/TGA (H3)	TGG/CGG (H4)	TGG/TCA (H5)	TGG/CCG (H6)	TCG/TCG (H7)	*p* value^b^
Women								
*N* (%)	198 (31.2)	146 (23.0)	96 (15.1)	43 (6.8)	37 (5.8)	35 (5.5)	36 (5.7)	
BMD TH (g/cm^2^)	0.80 (0.13)	0.81 (0.14)	0.80 (0.14)	0.78 (0.11)	0.78 (0.13)	0.83 (0.16)	0.76 (0.13)	0.149
BMD LS (g/cm^2^)	0.90 (0.17)	0.91 (0.16)	0.91 (0.17)	0.93 (0.12)	0.90 (0.16)	0.94 (0.19)	0.89 (0.17)	0.731
BMD FN (g/cm^2^)	0.66 (0.11)	0.67 (0.12)	0.66 (0.11)	0.66 (0.10)	0.64 (0.12)	0.71 (0.13)	0.64 (0.11)	0.115
Men								
*N* (%)	68 (34.0)	37 (18.5)	41 (20.5)	16 (8.0)	13 (6.5)	13 (6.5)	12 (6)	
BMD TH (g/cm^2^)	0.95 (0.15)	0.95 (0.12)	0.91 (0.18)	0.99 (0.15)	0.96 (0.17)	0.97 (0.15)	0.89 (0.18)	0.910
BMD LS (g/cm^2^)	0.98 (0.17)	1.02 (0.20)	0.93 (0.14)	1.02 (0.17)	0.98 (0.17)	0.97 (0.18)	0.98 (0.15)	0.385
BMD FN (g/cm^2^)	0.76 (0.13)	0.75 (0.12)	0.74 (0.13)	0.79 (0.15)	0.81 (0.17)	0.78 (0.12)	0.73 (0.16)	0.903

*p* values are shown for analysis of covariance (ANCOVA) for the bone mineral density (BMD) parameters with age and BMI as covariates. Numbers are means (SD)

*LS* lumbar spine, *FN* femoral neck, *TH* total hip

^a^TGG/TGG indicates wild-type for Leu46Pro (TT), Arg312Ser (GG), and Arg334Cys (GG)

^b^Adjusted for age and BMI

No statistically significant differences were found between the common haplotype combinations and BMD values adjusted for age and BMI (Table 5). However, women heterozygous for the Leu46Pro (TC genotypes, i.e., haplotypes H4 and H6) showed increased BMD values at the lumbar spine compared to women wild-type for the Leu46Pro polymorphism (i.e., haplotypes H1–H3, H5, and H7) (BMD_LS_ = 0.93–0.94 vs 0.89–0.91, respectively). Haplotype H6, which, besides Leu46Pro, also includes individuals heterozygous for the Arg334Lys SNP (GC), also showed increased BMD values at the hip, compared to haplotypes in which women were wild-type for the Leu46Pro polymorphism (i.e., haplotypes H1–H3, H5, and H7) (BMD_TH_ = 0.83 vs 0.76–0.81 BMD_FN_ = 0.71 vs 0.64–0.67, respectively). Using a model in which haplotypes H1–H3, H5, and H7 were combined, this increase in femoral neck BMD was statistically significant (*p* = 0.04).

## Discussion

Within a cohort of Dutch fracture patients, we have shown that the Leu46Pro polymorphism in the P2Y_2_ receptor gene was associated with increased BMD values in women, i.e., decreased risk of osteoporosis. This result is consistent with our main hypothesis that genetic aberration of P2Y_2_ receptor function affects BMD in humans. However, both the Arg334Cys and Arg312Ser polymorphisms had no significant relationship with BMD in human.

This is the first study demonstrating an association between the Leu46Pro polymorphism and osteoporosis risk. Women homozygous for the variant allele of the Leu46Pro polymorphism had significantly increased BMD values and a decreased risk of osteoporosis. So far, no in vitro studies have established the effect of this polymorphism on P2Y_2_ receptor function as well as on bone cell function. Since in vitro experiments and animal studies failed to clarify whether the P2Y_2_ receptor plays an anti- or pro-osteogenic role in bone, it remains unclear at present whether the Leu46Pro polymorphism is a loss- or gain-of-function SNP. Cell studies on the functional effect of this polymorphism are therefore warranted.

The Arg334Cys and Arg312Ser polymorphisms had no significant relationship with BMD in our study population. Both these polymorphisms have been shown to increase P2Y_2_ receptor function in vitro, as cells homozygous for the variant allele showed significantly increased transient Ca^2+^ influx compared to wild-type cells [19, 20]. Our findings showed that women homozygous for the variant allele of the Arg334Cys or Arg312Ser polymorphism had decreased BMD values compared to women carrying at least one wild-type allele, though not statistically significant. Recently published preliminary data from a Danish cohort consisting of 2,016 early postmenopausal women, however, showed that women homozygous for the variant allele of the Arg312Ser polymorphisms had significantly increased hip BMD at menopause (mean BMD ± SEM, 0.94 g/cm^2^ ± 0.012 (*p* = 0.017)) compared to women with wild-type allele (0.92 g/cm^2^ ± 0.004) after correction for age and logBMI. Furthermore, they found that women with wild-type allele had almost 25 % higher rate of bone loss at the lumbar spine at both 5 and 10 years after menopause than women homozygous for the variant allele (respectively: 5 years, −1.23 and −1.01 % per year (*p* = 0.008); 10 years, −0.76/−0.62 % per year (*p* = 0.028)) [22]. Therefore, larger association studies will be needed to elucidate the association between the Arg312Ser polymorphisms and BMD values.

The analysis of haplotypes showed that women carrying the variant allele of both the Leu46Pro and Arg312Ser polymorphisms had increased BMD values at all sites. As the analyses of individual SNPs showed increased BMD values in women carrying at least one variant allele of the Leu46Pro, whereas women carrying at least one variant allele of the Arg312Ser showed decreased BMD values, it can be speculated that a presumed loss of P2Y_2_ receptor function due to the presence of the Leu46Pro polymorphism has a dominant positive effect on BMD if expressed together with the variant allele of the Arg312Ser polymorphism.

It should be noted that, although the allele frequencies of the studied non-synonymous SNPs in our population were comparable to previously published data [19, 20, 22], both the Leu46Pro and Arg334Cys polymorphisms showed significant deviation from HWE. Therefore, studies in different populations are needed to confirm our findings.

Our study has several limitations. One limitation is the possibility of false negative findings due to lack of power, as is the case in many other genetic association studies. A second limitation of our study was that we did not have access to reliable information on other risk factors for osteoporosis, such as vitamin D and calcium intake, physical activity, and years since menopause, so that our analyses could not be adjusted for these factors. However, unlike classical epidemiological studies, genetic association studies are unlikely to be confounded by behavioral and environmental factors as these factors are very unlikely to show an association with the genotype. We therefore think that confounding by these risk factors or other unmeasured factors was not an issue. Nevertheless, the lack of adjustment might have influenced the precision of our results. Furthermore, exploration of the presence of gene–environment interactions was limited by the lacking information on certain osteoporosis risk factors.

We deliberately chose a fracture cohort with high prevalence of osteoporosis to investigate the associations between P2Y2 SNPs and osteoporosis risk for reasons of efficiency. Although it is a limitation that this cohort is not population based, it should be noted that for genetic screening purposes, such a high-risk population is considered more relevant than a general population.

Recently performed genome-wide-association studies (GWAS) have confirmed many previously identified candidate genes for osteoporosis, such as LRP5, OPG, RANK, and RANKL [23], and polymorphisms affecting the expression of these gene products have implications on bone mass and strength. However, the effect sizes are relatively small in a polygenetic trait such as BMD. Nevertheless, current GWAS studies are best powered for SNPs with a population frequency in the range of 10–90 %. Therefore, relatively rare polymorphisms such as the Leu46Pro polymorphism, which has a population frequency around 9 % in Caucasian, would likely have been missed in GWAS studies.

In conclusion, this is the first study describing an association between the Leu46Pro polymorphism in the P2Y_2_ receptor gene and BMD, supporting a role for this gene in the regulation of human bone mass. More studies are warranted to elucidate the exact role of the P2Y2 receptor in the bone physiology.
